# Catalytic Stability of *S*-1-(4-Hydroxyphenyl)-Ethanol Dehydrogenase from *Aromatoleum aromaticum*

**DOI:** 10.3390/ijms25137385

**Published:** 2024-07-05

**Authors:** Mateusz Tataruch, Viera Illeová, Anna Kluza, Patrik Cabadaj, Milan Polakovič

**Affiliations:** 1 Jerzy Haber Institute of Catalysis and Surface Chemistry Polish Academy of Sciences, Niezapominajek 8, 30-239 Krakow, Poland; mateusz.tataruch@ikifp.edu.pl (M.T.); anna.kluza@ikifp.edu.pl (A.K.); 2 Institute of Chemical and Environmental Engineering, Slovak Technical University, Radlinského 9, 812 37 Bratislava, Slovakia; viera.illeova@stuba.sk (V.I.); patrik.cabadaj@stuba.sk (P.C.)

**Keywords:** *S*-1-(4-hydroxyphenyl)-ethanol dehydrogenase, inactivation, aggregation, thermal inactivation mechanism, enzyme stabilization

## Abstract

Derived from the denitrifying bacterium *Aromatoleum aromaticum* EbN1 (*Azoarcus* sp.), the enzyme *S*-1-(4-hydroxyphenyl)-ethanol dehydrogenase (S-HPED) belongs to the short-chain dehydrogenase/reductase family. Using research techniques like UV-Vis spectroscopy, dynamic light scattering, thermal-shift assay and HPLC, we investigated the catalytic and structural stability of S-HPED over a wide temperature range and within the pH range of 5.5 to 9.0 under storage and reaction conditions. The relationship between aggregation and inactivation of the enzyme in various pH environments was also examined and interpreted. At pH 9.0, where the enzyme exhibited no aggregation, we characterized thermally induced enzyme inactivation. Through isothermal and multitemperature analysis of inactivation data, we identified and confirmed the first-order inactivation mechanism under these pH conditions and determined the kinetic parameters of the inactivation process. Additionally, we report the positive impact of glucose as an enzyme stabilizer, which slows down the dynamics of S-HPED inactivation over a wide range of pH and temperature and limits enzyme aggregation. Besides characterizing the stability of S-HPED, the enzyme’s catalytic activity and high stereospecificity for 10 prochiral carbonyl compounds were positively verified, thus expanding the spectrum of substrates reduced by S-HPED. Our research contributes to advancing knowledge about the biocatalytic potential of this catalyst.

## 1. Introduction

Enantioselective alcohol dehydrogenases are considered very versatile and valuable biocatalysts. They are characteristic by high stereoselectivity and the ability to catalyze both the oxidation of alcohols and the reduction of carbonyl compounds. Moreover, NAD(P)-dependent dehydrogenases are popular biotools for the biotechnological synthesis of chiral compounds [1,2,3,4,5,6,7,8].

The *A. aromaticum* EbN1 bacterium is the source of unique and interesting enzymes for researchers. Thanks to these enzymes, the bacterium is capable of anaerobic and aerobic degradation of 47 organic growth substrates [9]. These enzymes include, among others, biocatalysts with catalytic transition metals (metalloenzymes), such as molybdenum-dependent ethylbenzene dehydrogenase (EBDH) [10,11,12,13,14] and tungsten-dependent aldehyde oxidoreductases (AOR) [15,16,17,18]. Additionally, there are metal-independent oxidoreductases from the short-chain dehydrogenase/reductase (SDR) family, such as (*S*)-1-phenylethanol dehydrogenase (S-PEDH) [1,19,20] and *R*-/*S*-1-(4-hydroxyphenyl)-ethanol dehydrogenase (R-HPED and S-HPED) [21,22,23,24,25].

S-HPED is encoded by the *ebA309* gene and, from a stereoselectivity standpoint, it acts as the mirror enzyme for the R-HPED whose gene, *ebA307* (*chnA*), is located in the same operon *eph* [24]. In terms of the Prelog rule, S-HPED has been characterized, in contrast to R-HPED, as an anti-Prelog dehydrogenase [23]. S-HPED participates in the anaerobic mineralization pathway of p-ethylphenol in the denitrifying bacterium *A. aromaticum* EbN1. It was initially suspected to be involved in the second step of p-ethylphenol mineralization, specifically in the dehydrogenation of 1-(4-hydroxyphenyl)-ethanol to 4-hydroxyacetophenone [25,26]. However, Büsing et al. showed that S-HPED is not responsible for this NAD^+^-dependent oxidation of 1-(4-hydroxyphenyl)-ethanol [23]. Instead, R-HPED was identified as the enzyme carrying out this function [23]. Although the precise metabolic role of S-HPED in this pathway remains unknown, the latest reports reveal the practical possibilities of its use. Wu et al. recently presented the results of studies optimizing the conditions of 4-hydroxyacetophenone biosynthesis using S-HPED [21] as a promising alternative to plant extraction or chemical synthetic approaches [27,28,29]. In this case, the oxidative catalytic activity of these enzymes was utilized. Thus, available preliminary reports on this enzyme, its reactivity and stereoselectivity, indicate that it may be a biocatalyst with great, although still untapped potential, and therefore constitute a promising biotool competing with the well-characterized S-PEDH from *A. aromaticum* from the ethylbenzene metabolic pathway [21].

As with any biocatalyst, the use and storage of S-HPED are burdened by the problem of its catalytic instability. Due to the complexity of their structure, biocatalysts are unstable, which results in a progressive loss of their activity. Therefore, besides selectivity, the robustness of a biocatalyst against inactivating conditions during reactions and storage is one of the most desired properties. In particular, resistance to thermal inactivation and inactivation induced by extreme pH and components of the reaction or storage environment is important for the practical use of enzymes [3]. Currently, modern genetic engineering methods make it possible to design in silico changes in the amino acid structure of a protein, which can potentially increase the thermal stability of the enzyme. The success in stabilizing alcohol dehydrogenases based on the in silico method and amino acid engineering was presented by, among others, Fraaije’s [30] and Qu’s [31] teams. However, despite the progress related to the use of protein genetic engineering methods, classic methods of improving the stability of enzymes and biocatalytic processes are still used. Using classic approaches to stabilization, such as immobilization or reaction environment engineering methods, it is possible to significantly prevent the inactivation of the biocatalyst and improve its storage and process stability and efficiency [32,33,34,35,36].

The catalytic stability of R-HPED under various thermal and pH conditions and the mechanism of enzyme inactivation at alkaline pH were described in our previous article [22]. In this article, we provide the basic characteristics of S-HPED, and, more importantly, results that identify factors affecting the enzyme’s deactivation. We present the dynamics of the enzyme activity loss process during the reaction and during enzyme storage, induced by pH and temperature and in the presence of glucose, which is a protein stabilizer. We also put forward and discuss the proposed mechanism of S-HPED inactivation during storage under alkaline conditions. Information presented herein will be necessary to conduct further applied research on S-HPED and ultimately for its practical use in industrial syntheses.

## 2. Results and Discussion

### 2.1. Synthesis of Chiral Alcohols with Pure S-HPED

Büsing et al. pointed out S-HPED’s potential for producing enantiomerically pure secondary alcohols from a wide range of prochiral ketones and esters [23]. Moreover, they investigated and positively confirmed that S-HPED reduces compounds like 4′-hydroxyacetophenone, 2-chloro-4′-hydroxyacetophenone, 2-chloroacetophenone and acetophenone. Borowiecki et al. positively verified S-HPED’s activity for methyl 4-oxo-4-phenylbutanone [2]. Taking into account the extensive substrate spectrum identified for well-studied S-PEDH from *A. aromaticum* [1], we decided to search for additional carbonyl substrates that are reduced by S-HPED. We checked the S-HPED activity of another 15 prochiral compounds and related their initial activity to the activity of acetophenone. For 11 out of 15 investigated ketones, S-HPED exhibited reducing activity (Table 1). Activity assay with 1-indanone-derived substrates (1-indanone, 2,3-benzofuran, 3-phenyl-1-indanone and 6-hydroxy-1-indanone) that were reduced in the presence of S-PEDH showed no S-HPED activity. For the most active substrates, only one enantiomeric form of the alcoholic product was detected, which has been proven by chiral chromatography analysis. We can infer that the substrate spectrum of S-HPED is wide, similar to that of S-PEDH. For 4′-aminoacetophenone, two product peaks were identified, with retention times corresponding to those observed for the *S*- and *R*-1-(4-aminophenyl)ethanol forms by Dudzik et al. [1]. It is noteworthy that the S form of the product predominated, contrasting with the racemic product obtained from the reaction catalyzed by S-PEDH [1], which indicates the complementary nature of these enzymes with certain substrates.

### 2.2. Effects of pH and Reaction Temperature on the Activity of S-HPED

**Figure 1:**
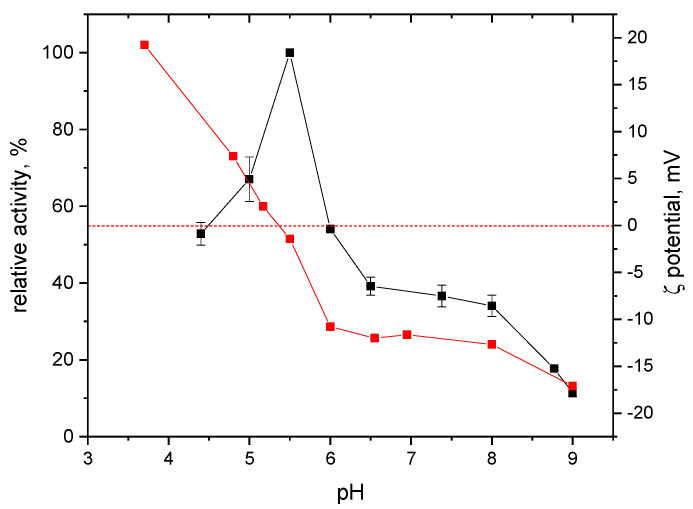
Effect of pH on the S-HPED activity (black squares) and zeta potential derived from the electrophoretic mobility (red squares).

A series of tests were performed to determine the basic properties of the biocatalyst, including its activity as a function of pH and temperature and its isoelectric point. The effects of pH on reductive activity were measured in a pH range of 4.4 to 9.0. The enzyme exhibited maximum reductive activity around pH 5.5 (Figure 1), which closely aligns with the optimum pH of R-HPED [22]. Minimal reductive enzyme activity was observed at pH 9.0.

**Figure 2:**
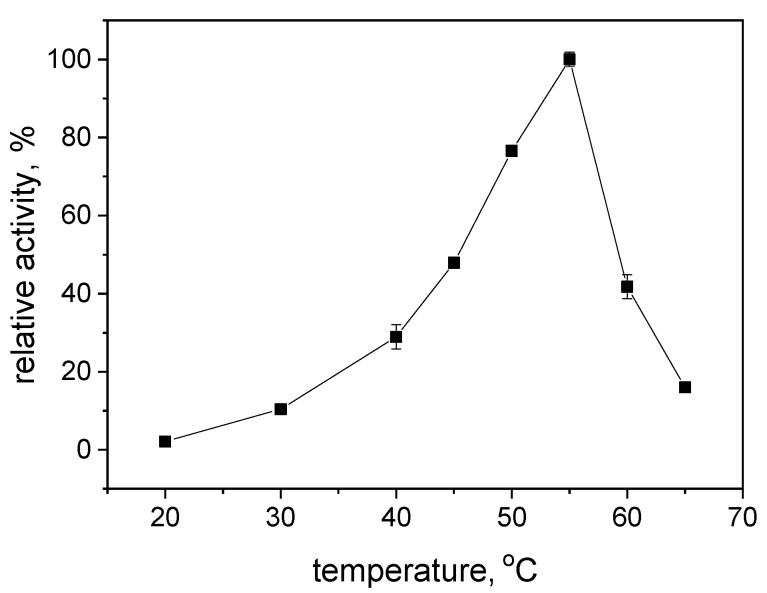
Effect of temperature on the activity of S-HPED.

The optimum temperature was found to be 55 °C (Figure 2). Additionally, the determined isoelectric point of the protein was measured at 5.3 (Figure 1), coinciding with the pH range where the enzyme exhibits maximum initial activity. This observation aligns with the relationship between activity and isoelectric point observed for the analogous R-HPED enzyme [22].

To determine the conditions conducive to S-HPED long-term stability, the influence of temperature and pH during enzyme incubation on the activity of the enzyme was examined. Initially, a series of tests was carried out at a relatively low temperature of 20 °C.

**Figure 3:**
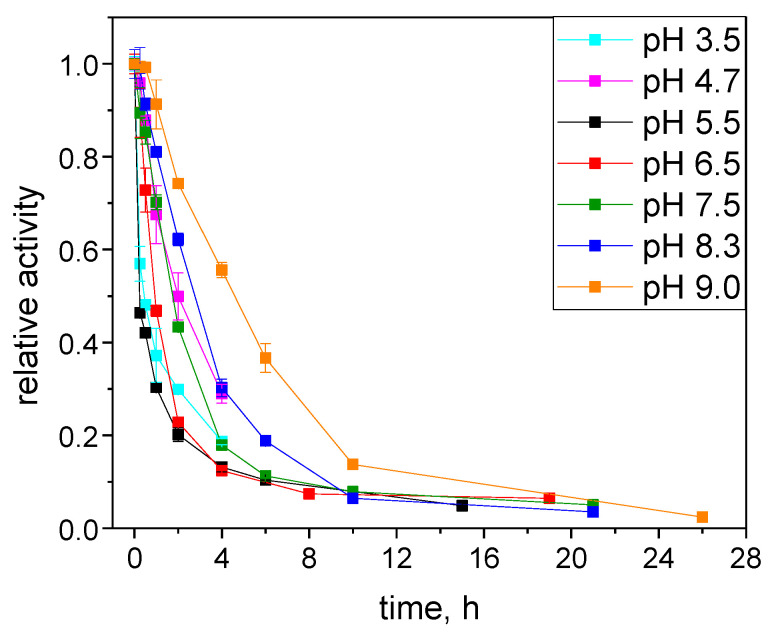
The pH stability of S-HPED stored at temperature 20 °C.

Figure 3 presents the loss of S-HPED activity for the enzyme incubated at various pH at 20 °C where thermal inactivation plays a minor role compared to pH-induced inactivation. Therefore, the observed dynamics of the activity loss mainly reflect the impact of the pH as an inactivation factor. Similarly to R-HPED inactivation [22], the fastest loss of activity, was observed at pH 5.5 where the initial activity of the enzyme is the highest (Figure 1). At pH 5.5, S-HPED loses almost 90% of its activity within just 4 h. Interestingly, the inactivation rate at pH 4.7 was slightly lower and comparable to that at pH 6.5 and 7.5. This may be due to increased repulsive interactions of positively charged protein molecules (Figure 1) counteracting the effect of acid inactivation.

The latter effect became more pronounced at the lower pH of 3.5, resulting in approximately the same inactivation rate as at pH 5.5. The stabilizing effect of higher pH is best demonstrated at pH 9.0 where the enzyme loses 90% of its activity after 10 h. However, the stability of S-HPED at pH 9.0 is much lower than that of R-HPED, which was stable at pH 8.5 for 8 days [22]. Despite the high initial activity of the enzyme at pH 5.5, the isoelectric point region determines its biochemical properties at this pH. A pH close to the isoelectric point induces rapid protein inactivation, which is putatively affected by rapid aggregation of the enzyme due to the reduction of repulsive electrostatic interactions between protein molecules.

#### 2.2.1. Impact of Glucose

**Figure 4:**
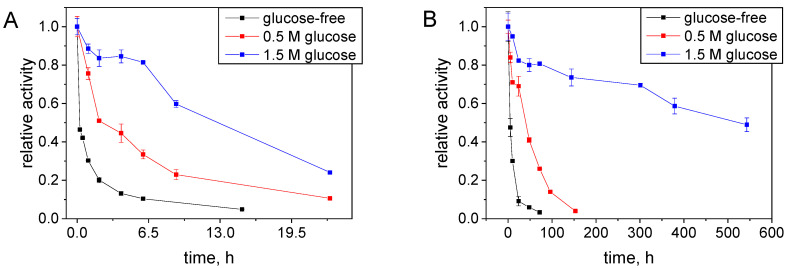
S-HPED stability (**A**) at pH 5.5 and (**B**) at pH 9.0 at 20 °C in the presence of various concentrations of glucose.

One of the factors that stabilize enzymatic activity is the presence of sugars. The theoretical justification for this phenomenon could be based on vitrification theory which describes enzyme stabilization from a kinetic perspective [37]. The mechanism of protein stabilization by sugars against thermal stress relies on forming an immobilizing matrix around the protein, which ultimately reduces local and global mobility, resulting in protein structure preservation [37]. Based on our previous studies on R-HPED under storage conditions [22] indicating a high increase of protein stability in the presence of glucose, the effect of glucose addition on the rate of S-HPED inactivation was examined. Figure 4 compares the dynamics of the S-HPED activity decrease when the enzyme is stored at 20 °C under two boundary pH values 5.5 and 9.0 in the presence of various glucose concentrations. It shows that as the sugar concentration increases, the rate of enzyme inactivation slows down. The highest stabilizing effect was observed for the highest glucose concentration of 1.5 M. At pH 9.0, under this concentration, the initial activity of the enzyme decreased by 50% over 690 h, while in the glucose-free buffer, the activity dropped below 10% in less than 24 h. A significant stabilizing effect of sugar was also observed at pH 5.5 (Figure 4A), where the enzyme rapidly aggregates and loses activity.

#### 2.2.2. Thermal Shift Assay

**Figure 5:**
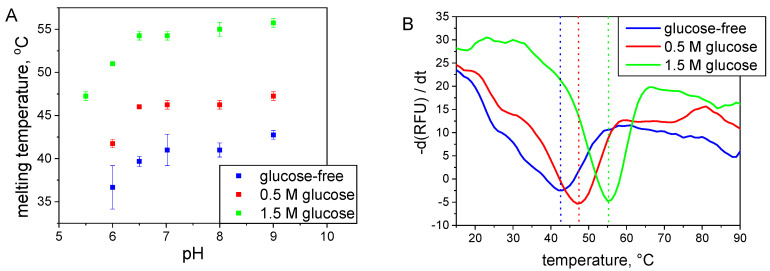
TSA measurements results. (**A**) Plot of T_m_ values of S-HPED versus pH at different glucose concentrations. (**B**) Illustrative TSA melting curves of S-HPED obtained in 100 mM Tris/HCl pH 9.0 and exhibiting T_m_ values 42.8 °C, 47.3 °C and 55.8 °C for buffer with 0 M, 0.5 M and 1.5 M of glucose, respectively.

The thermal shift assay (TSA) technique identifies the thermal denaturation temperature (melting temperature) of the protein under various conditions. We examined the temperature-induced melting profiles of S-HPED in various buffers and 0.5 and 1.5 M glucose at pH values in the range of 5.5–9.0, (Figure 5). At the lowest pH of 5.5, rapid aggregation hindered the determination of the protein denaturation temperature without a sufficient addition of glucose stabilizer. The lowest thermal stability was observed for the buffer solutions without glucose. The addition of glucose causes a significant increase in the melting point, which remains consistent across the entire tested pH range. Specifically, for the solutions containing 0.5 and 1.5 M glucose, this increase amounts to 5.2 ± 0.6 °C and 13.5 ± 0.9 °C, respectively. In all solutions, the melting temperature has the highest value between pH 7.0 and 9.0. At lower pH values, the melting temperature was up to approximately 5 °C lower.

### 2.3. Catalytic Stability and Aggregation of S-HPED at Elevated Temperatures

#### 2.3.1. Catalytic Stability Study under Storage Conditions

The dynamics of inactivation and aggregation of the enzyme was further examined at an elevated temperature of 45 °C, at which the enzyme possesses good activity (Figure 2) and which could be pertinent to reactor applications. The results obtained are presented in Table 2. In the entire range of the tested pH, the activity of the enzyme without additives and with the addition of acetophenone decreased within the first 30 min of incubation to a value below 3% of the initial activity. At this temperature, 30 min is too long to clearly differentiate the stability of the enzyme by pH. The presence of the substrate, acetophenone (2 mM), which could potentially protect the enzyme by stabilizing its active site, did not improve the stability of the enzyme over 30 min. Only the enzyme incubated at 45 °C in the presence of 1.5 M glucose showed an improvement in stability, retaining, depending on pH, from 9% to 60% of its initial activity after 30 min of incubation, up to 15% of activity after 2 h and residual activity after 5 h of incubation.

#### 2.3.2. Catalytic Stability Study under Reactor Conditions

**Figure 6:**
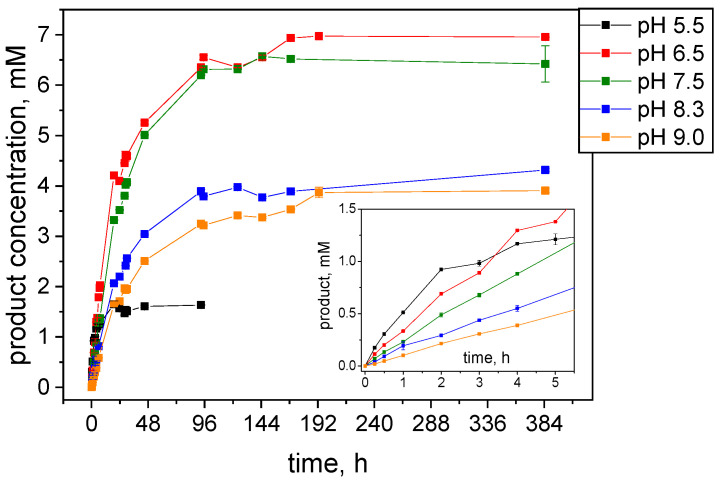
Progress curves of acetophenone conversion to (*S*)-1-phenylethanol by S-HPED conducted at different pHs. The inset depicts the product concentration changes during the initial 5 h of reaction.

The stability of the enzyme at 45 °C in reactor conditions significantly increased, with the enzyme remaining active for over 48 h (Figure 6). The curves depicting the growth of product concentration during reactions in NADH recovery mode reflect the relative rate of enzyme inactivation under conditions that vary only in the pH of the reaction mixture. The increase in product concentration during the initial phase of the reaction (first two hours) is most rapid at pH 5.5 and slows down as the pH increases (Figure 6, inset), consistent with the determined maximum of biocatalyst activity (Figure 1). However, in a more alkaline medium, the stability of the enzyme increases. Thus, the maximum product yield obtained at different reaction pH is influenced by both the reaction rate and the deactivation rate, with the highest in the pH range of 6.5–7.5. At pH 5.5, the reaction progressed for 24 h, ultimately reaching a product concentration of 1.6 mM. The highest product concentration, 7 mM, was achieved after 7 days of reaction at pH 6.5. This efficiency indicated that pH 6.5 provides the best compromise between enzyme activity and stability in investigated pH range.

#### 2.3.3. Aggregation under Storage Conditions

**Figure 7:**
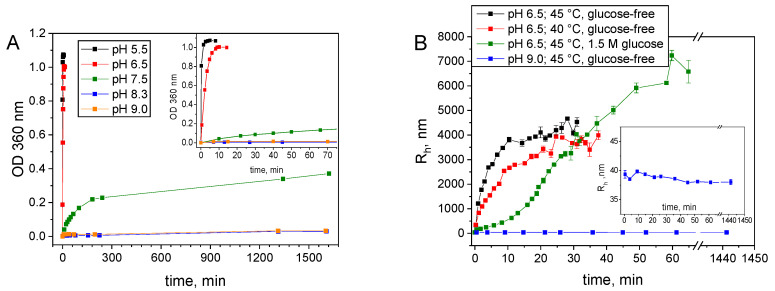
Dynamics of S-HPED aggregation at different pH. (**A**) OD_360nm_ courses at different pH at 45 °C. The inset plot presents the aggregation courses during the initial period. (**B**) Courses of hydrodynamic radius R_h_ at different conditions. The inset shows a magnification of the R_h_ values at pH 9.0.

The aggregation rate of S-HPED at 45 °C increased as the pH of the environment decreased, with the fastest aggregation observed at pH 5.5 (Figure 7A). Conversely, at pH 8.3 and 9.0, aggregation was practically undetectable even after 15 h, as indicated by the turbidimetric method, despite a complete loss of enzyme activity within 30 min. Figure 7B presents the results of dynamic light scattering (DLS) measurements conducted at pH 9.0 and 6.5, and at temperatures of 40 °C and 45 °C, respectively. The hydrodynamic radius values document no appearance of aggregates during several days of incubation of the protein at pH 9.0, while a rapid increase of aggregate size is observed at pH 6.5. Additionally, the growth of aggregates is significantly slower in the presence of 1.5 M glucose. The above observations lead us to the hypothesis that, similar to R-HPED, at alkaline pH far from the isoelectric point of S-HPED, the process of protein activity loss caused by temperature is not affected by aggregation. To confirm this assumption, the next phase of the research involved conducting a series of thermal inactivation experiments at pH 9.0. The obtained data underwent statistical analysis to study the temperature stability of S-HPED in detail and determine the mechanism of thermal inactivation. Given the structural similarity between S-HPED and R-HPED, the analogue that we previously tested and the absence of S-HPED aggregation at pH 9.0, it is suggested that the most likely inactivation mechanism follows an irreversible one-step transition of the active enzyme form into an inactive one described by (Equation (1)).

### 2.4. Modelling of Thermal Inactivation under Storage Conditions

The assessment of experimental data compliance with the “one-step, two-states” mechanism was carried out using the method of isothermal fitting, which was subsequently validated by multi-temperature fitting. The assumptions of these two approaches are described in [22], where we examined the mechanism of thermal inactivation of R-HPED, as well as in the numerous works on enzyme inactivation mechanism modelling [38,39,40,41,42].

**Figure 8:**
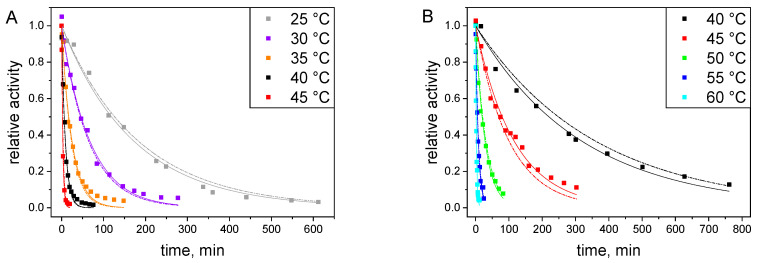
Thermal inactivation of S-HPED at pH 9.0 (**A**) without additives and (**B**) in 1.5 M glucose. The symbols represent experimental data at different temperatures; the lines represent fits with the first-order model using isothermal evaluation (solid lines) and multitemperature evaluation (dashed lines).

Figure 8 depicts model fits to the experimental results obtained for inactivation in the presence of glucose and glucose-free buffer using isothermal (solid lines) and multitemperature (dashed lines) evaluations. Table 3 and Table 4 present the *k* values obtained from the isothermal evaluation and the estimated values of parameters *k*_0_ and *E*_a_ from the multi-temperature evaluation, respectively.

**Figure 9:**
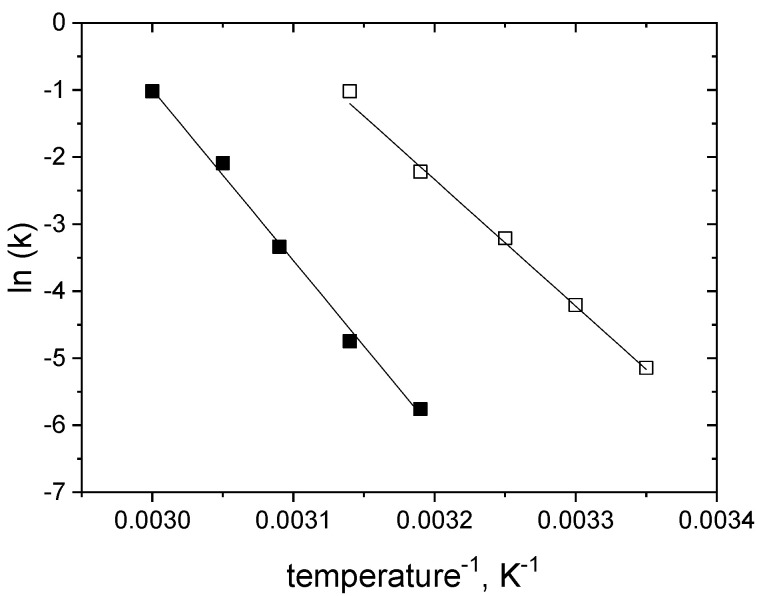
Arrhenius plots for the first-order rate constants of S-HPED inactivation at pH 9.0. The symbols represent the values of k obtained by isothermal evaluation at the different conditions: empty squares—glucose-free; full squares—in 1.5 M glucose (Table 3). The straight lines represent the courses calculated from the coefficients of the Arrhenius equation obtained by the multitemperature evaluation (Table 4).

In the isothermal approach, the standard deviations of activity for each temperature did not exceed the limit value from the 95% confidence interval; therefore, the assumed model was accepted as adequate to represent enzyme activity decays at all temperatures studied. The fact that the first-order mechanism correctly describes the inactivation data was further confirmed by meeting this adequacy criterion also in the multitemperature evaluation (Figure 9).

The glucose-induced improvement of enzyme stability under the storage regime was confirmed by a decrease in the k values at temperatures 40 and 45 °C (Table 3). The inactivation rate constants of S-HPED in the presence of glucose were approximately 30 to 40 times lower than the corresponding values in glucose-free conditions. Based on this difference, we can infer that in the case of S-HPED, the stabilizing effect of glucose is over 15 times greater than for R-HPED, for which, when using the same amount of glucose, k decreased by about 2.5 times.

The phenomenon of protein stabilization by glucose can be explained by the vitrification theory. According to this theory, the stabilization effect is based on forming a liquid glass matrix around the protein. When the enzyme is entrapped in this saccharide-glass matrix, the matrix reduces the mobility of protein particles, which causes the sugar matrix to protect the enzyme structure against thermal stress.

Comparing the values of *E*_a_ obtained for both inactivation conditions (Table 4), we observe a difference of 35%, while for R-HPED these activation energy differences were negligible. Nonetheless, it can be concluded that the stabilizing effect of glucose primarily has an entropic character, which is another analogy to R-HPED [22].

Despite the low activity of S-HPED and the low reaction efficiency under alkaline pH, its relatively high thermal stability under these conditions and the lack of aggregation indicate that alkaline pH is a favorable environment for storing the enzyme. Moreover, the previously discussed absence of aggregation at pH 9.0 could serve as a premise for stabilizing S-HPED through its covalent immobilization in this pH environment. Subsequently, the immobilized enzyme would be tested under process conditions across a wider pH range, including pH 5.5, where its initial activity is the highest.

Comparison of the results of the aggregation kinetics and activity loss kinetics studies indicates that the optimal pH environment in which the enzyme loses activity at the slowest rate under storage conditions is an alkaline environment (pH 9.0 in the pH range tested). In such an environment, the dynamics of activity loss is the slowest and, due to the significant distance from the isoelectric point, aggregation does not occur at all. In addition, the enzyme stored in such conditions has a relatively high melting point. The stability of the enzyme is significantly improved by enriching the storage medium with sugar. A 1.5 molar addition of glucose at pH 9.0 significantly increases the melting point by 13 °C and reduces the enzyme inactivation rate constant by about 35 times.

The optimum reaction pH, on the other hand, is an environment within the pH range of 6.5, where the efficiency of the biocatalyst is relatively highest. The phenomenon of highest efficiency we observe at pH 6.5 may be caused by the superposition of stability and activity of the enzyme in these environments.

**Figure 10:**
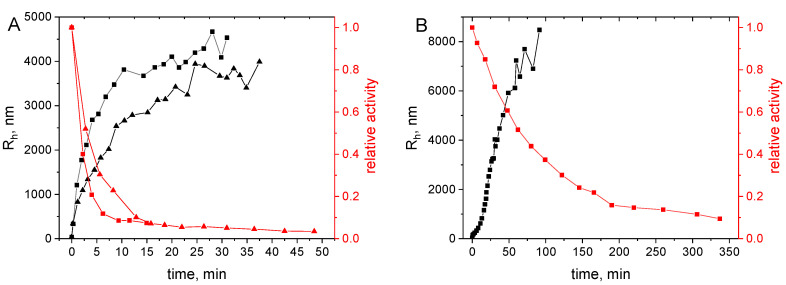
Juxtaposition of the dynamics of inactivation (red points) and aggregation (black points) for S-HPED at pH 6.5 and the temperature of 40 °C (triangles) and 45 °C (squares), respectively. (**A**) glucose-free solution, (**B**) in 1.5 M glucose.

Thermal inactivation at a lower pH such as 6.5, which is closer to the isoelectric point of the protein, may be augmented by an additional inactivation mechanism, namely aggregation. It is likely that due to this factor, the mechanism of S-HPED inactivation under lower pH conditions could be more intricate. It is noteworthy that at pH 6.5, enzyme activity persisted despite the presence of protein aggregates at an intermediate stage of inactivation and even after prolonged exposure of the protein to elevated temperatures (Figure 10). These observations provide an opportunity for further exploration regarding the influence of the aggregation process on the activity loss at pH 6.5. On one hand aggregation, may act as an additional factor leading to inactivation, but, on the other hand, the aggregated form may offer partial stabilization to the biocatalyst.

The juxtaposition and analysis of the relationship between the dynamics of aggregation and the dynamics of inactivation for S-HPED, similar to that for R-HPED discussed in our previous work [22], provide the basis of our hypothesis. We propose that fast aggregation in the pH region near the isoelectric point (pI) is a process that modifies the basic, first-order mechanism of thermal inactivation for both S- and R-HPED within this pH range. At a pH far from the pI, aggregation does not occur at all, or it is such a slow process that it is not measurable within the time frame of complete enzyme inactivation. Under these conditions, the inactivation of the biocatalyst occurs solely due to the thermal factor, following the first-order mechanism, as observed in the case of S-HPED.

As the pH approaches the pI, the aggregation dynamic becomes faster. In the pH region slightly closer to the pI, aggregation becomes observable and clearly measurable within the inactivation time frame. Despite this, aggregation remains a non-dominant process and is slower than thermal inactivation. Therefore, even with the clear presence of aggregated protein in the solution, primary inactivation mechanism is still first-order, and aggregation likely occurs in already deactivated catalyst molecules. This case was also reported for R-HPED [22]. A change in the inactivation mechanism can occur putatively only in a pH range very close to the pI, where phenomena induced by aggregation—such as catalytic stabilization by shielding protein molecules inside the aggregate and diffusion limitations—begin to modify the dynamics of the first-order activity loss process.

## 3. Materials and Methods

### 3.1. Chemicals

Culture media: LB Broth media from BioShop^®^ Canada (Burlington, ON, Canada), anhydrotetracycline hydrochloride from Supelco (Bellefonte, PA, USA). Purification: d-desthiobiotin from and 2-(4-hydroxyphenylazo)benzoic acid (HABA) from Sigma-Aldrich (Saint-Louis, MO, USA). Tested substrates: acetophenone 99%, 4′-chloroacetophenone 98+%, 4′-metoxyacetophenone 99%, 4′-aminoacetophenone 99%, 4-acetylbenzonitrile 99% were purchased from Alfa Aesar while 4′-fluoroacetophenone 99%, 4′-nitroacetophenone 98%, 3′-aminoacetophenone 97%, 4′-ethylacetophenone 97%, 4′-bromoacetophenone 98%, 1-indanone 99%, 2,3-benzofuran 99%, 3-phenyl-1-indanone 98%, 6-hydroxy-1-indanone 97% were purchased from Sigma-Aldrich. Activity assay: β-Nicotinamide adenine dinucleotide (NADH), Tris buffer, MES buffer, D-(+)-glucose were purchased from Sigma-Aldrich. TSA measurements: SYPRO Orange protein-dye was purchased from Life Technologies (Carlsbad, CA, USA). HPLC analysis: acetonitrile, n-hexane and isopropanol were purchased from Chemsolve^®^ (Roanoke, VA, USA).

### 3.2. Preparation of S-HPED

S-specific 1-(4-hydroxyphenyl)-ethanol dehydrogenase (Gene ID: ebA309) with N-terminal Strep-tag was overexpressed in *Escherichia coli* DH5α cells as previously described [24]. After cell disruption by sonication, the cell debris was removed by ultracentrifugation (100,000 g) and the recombinant protein was purified via Strep-tag affinity chromatography according to the supplier’s recommendation (IBA GmbH, Gottingen, Germany).

### 3.3. Synthesis of Chiral Alcohols with Pure S-HPED

To assess the enantioselectivity of the enzyme, the reactions with 15 representative keto substrates were performed. The reactions were conducted at 45 °C in 5 mL of 100 mM Tris/HCl buffer (pH 7.5) containing 1 mM NADH and 5 μL purified S-HPED (3.4 mg mL^−1^). The reactions’ start was initiated by the addition of 50 μL of a respective substrate stock solution in isopropanol (IPA). The reactions were stopped after 15 h of incubation, and the analytes were extracted from the water phase by solid phase extraction using Strata-X SPE columns from Phenomenex (Torrance, CA, USA) (500 mg/3 mL polymeric reversed phase). The analytes were eluted with 0.5 mL of IPA. IPA extracts of reaction mixtures were analyzed by chiral liquid chromatography.

### 3.4. Chiral Chromatographic Analysis

The liquid chromatographic analyses were performed on an Agilent 1100 System LC with a diode-array detector (DAD). The qualitative chiral analyses were performed using CHIRALCEL^®^ OB-H column (Daicel, Osaka, Japan, 250 × 4.6 mm, 5 μm) at 25 °C and n-hexane/IPA as mobile phase at a flow rate of 0.5 mL min^−1^ with different isocratic programs, depending on the substance (Figures S1–S12 in the Supplementary Materials).

### 3.5. Effect of pH on the Initial Activity of S-HPED and Activity Decrease Rate

The dependence of initial activity on pH was evaluated in 0.1 M MES/KOH buffer (pH points: 4.4, 5.0, 5.5; 6.0, 6.5) and 0.1 M Tris/HCl buffer (pH points 7.4; 8.0, 8.8; 9.0). The highest activity value obtained at pH 5.5 was taken as 100%. The thawed enzyme solution was diluted 10-fold in a series of buffers at 20 °C, gently mixed and immediately taken for the direct spectrophotometric activity assay.

In order to investigate the dynamics of activity loss depending on the pH at which enzyme is suspended, the thawed enzyme solution was diluted 10-fold in different buffers kept at 20 °C: 0.1 M MES/KOH pH 5.5; 6.5 and 0.1 M Tris/HCl pH 7.5; 8.3; 9.0. Tubes with diluted enzyme were incubated in a thermostat at 20 °C. At certain time intervals, samples of the diluted enzyme were transferred from the thermostated tubes directly to the quartz cuvette where its activity was measured in spectrophotometric assay. The residual activity was determined relative to the enzyme activity measured immediately after enzyme dilution (time 0). The same experimental procedure was done with buffers preheated at 45 °C.

### 3.6. Effects of Temperature on the Activity of S-HPED

To obtain the optimum temperature of S-HPED reductive activity the activity test was performed at the temperature range of 20–65 °C in 0.1 M MES/KOH buffer pH 6.5 according to the procedure described in paragraph 3.7. The pH of the reaction buffer was adjusted individually for each temperature. Acetophenone and NADH were added to the preheated buffer from the stock solution prepared in isopropanol immediately before enzyme addition.

### 3.7. UV-Vis Activity Assay

Residual ketone reduction activity of S-HPED samples was assayed in 0.1 M MES/KOH pH 6.5 preheated to 30 °C in quartz cuvettes. The assay was initiated by the addition of the enzyme solution to the preheated MES buffer containing 0.5 mM NADH and 2 mM acetophenone. The NADH oxidation was followed at 365 nm (ε = 3.4 mM^−1^ cm^−1^) using an Agilent 8453 UV/VIS spectrophotometer [1]. The activity was obtained from a linear fit to the first 15 s of the curve. All measurements were conducted in duplicate.

### 3.8. S-HPED Stability Tests under Reaction Condition

All reactions performed to investigate the impact of pH on S-HPED stability under reaction conditions were conducted in glass reactors under stirring mode with using magnetic stirrer (600 rpm) at a temperature 45 °C (water bath) in 10 mL of reaction mixture volume containing 10% of isopropanol and 90% of water buffer (100 mM MES/KOH buffer (pH 5.5 and 6.5) and 100 mM Tris/HCl buffer (pH 7.5, 8.3 and 9.0), pH of buffers was set at temperature 45 °C). Isopropanol was the second, alcohol substrate, used to carry out reactions under the NADH recovery regime. The initial concentrations of acetophenone substrate and NADH were 3.5 mM and 1.5 mM respectively. Reactions were initiated by adding 5 µL of S-HPED (3.44 mg mL^−1^) solution to the preheated reaction mixture solution. Samples of 20 µL were collected at selected time points and the enzyme was precipitated in 80 µL of ice-cold ACN. Every time the concentration of the substrate reached a concentration of approx. 1.5–2.0 mM a new portion of the substrate was injected into the reaction mixture. For each time point, HPLC analysis was performed in duplicates and average values were presented.

### 3.9. Chromatographic Analyses—Reverse Phase (RP-HPLC)

Concentrations of substrate (acetophenone) and product ((*S*)-1-phenylethanol) of the reaction were determined via HPLC using an Agilent 1100 system equipped with a DAD detector. The separations were performed on an Ascentic^®^ RP-Amide Express column (75 mm × 4.6 mm, 2.7 µm) at 40 °C with a water/ACN (65/35) mobile phase using an isocratic program at a flow rate of 1 mL min^−1^ and injection volumes of 5 µL. The quantitation of substrate and product was performed by integrating the peaks recorded by the DAD detector at 210 nm.

### 3.10. Aggregation Measurements

The aggregation of S-HPED in buffer solutions was observed at a temperature 45 °C as scattering at 360 nm [43] using the Agilent 8453 UV/VIS spectrophotometer (Santa Clara, CA, USA). To determine the dependence of aggregation rate on pH of the buffer in which the enzyme was suspended, a thawed enzyme solution was diluted 10 times (final concentration 0.344 mg mL^−1^) in a quartz cuvette with buffers pre-heated to 45 °C (0.1 M MES/KOH pH 5.5, 6.5 and 0.1 M Tris/HCl pH 7.5, 8.3, 9.0). The cuvette with diluted enzyme was thermostated at 45 °C in the Peltier cell holder controlling temperature or in an external thermostat.

### 3.11. Dynamic Light Scattering

The isoelectric point and dynamic of hydrodynamic radius change were measured using Malvern Zetasizer Ultra Red, ZS XPLORER 3.3.0.42 Software. The isoelectric point was determined from the curve of zeta-potential vs. pH of enzyme solution in 0.1 M MES/KOH pH 3.7–6.55 and 0.1 M Tris/HCl pH 6.95–9.0 at 20 °C (enzyme concentration 0.344 mg mL^−1^). Aggregation experiments were carried out under conditions analogous to the inactivation experiments. The average hydrodynamic radius of the enzyme particles (R_h_) was measured at 40 °C and 45 °C in 0.1 M MES/KOH pH 6.5 and 0.1 M Tris/HCl pH 9.0. Similar measurements were carried out at buffer pH 6.5 with 1.5 M of glucose. The pH values of buffers were adjusted at the proper temperature. In each experiment, the thawed enzyme solution was diluted 10-fold (final enzyme concentration 0.344 mg mL^−1^) in the appropriate, preheated buffer.

### 3.12. Thermal Shift Assay

The assay was carried out in a CFX Connect Real-Time PCR Detection System (BioRad, Hercules, CA, USA) using 96-well plates (#HSP9601, BioRad). Buffers MES/KOH and Tris/HCl in the pH range 5.5–9.0 were used for initial tests at 80 mM final concentration. In short, 20 μL of 100 mM selected buffer was added to the well followed by 2.5 μL of 500 times prediluted in water SYPRO Orange protein-dye (commercially available as stock solution 5000-times concentrate DMSO, #S6651, Life Technologies), to its final concentration of 1-fold (according to the manufacturer’s specification). The protein was thawed on ice and diluted directly into the wells of the PCR plate 10-times to the concentration of 0.344 mg mL^−1^ by adding 2.5 μL of concentrated protein (3.44 mg mL^−1^) to 22.5 μL of the respective buffer and Sypro Orange dye pre-mix. The assay was carried out using a temperature gradient starting from 10 °C to 95 °C with intervals of 15 s for each 1 °C. Thermal shift assays with glucose as an additive and selected buffers were performed analogously. Values of melting temperature (T_m_) were determined from the maximum of the first derivative taken from the plot of d(fluorescence)/dT vs. T of obtained raw fluorescence data. Measurements were done in triplicates or quadruplicates from which an average T_m_ was calculated.

### 3.13. Thermal Inactivation Experiments

Inactivation experiments for S-HPED were performed at pH 9.0 (100 mM Tris/HCl buffer) without glucose addition and with addition of 1.5 M glucose. 1.5 M glucose was used as an agent to prevent protein aggregation and inactivation during the inactivation experiment. The inactivation temperature range for condition without glucose was from 25 to 45 °C meanwhile for conditions with glucose temperature range was from 40 to 60 °C with an increment of 5 °C.

Inactivation experiments at each temperature were carried out as follows: thawed enzyme solution was diluted 10-fold (final enzyme concentration 0.344 mg mL^−1^) in 0.1 M Tris/HCl buffer pH 9.0 preheated to the specified temperature. The pH of the buffer was adjusted individually for each inactivation temperature. The diluted enzyme was gently mixed by pipetting and incubated in a thermostated tube. At different time intervals samples were taken, rapidly cooled in tubes immersed in an ice water bath and stored therein until the activity measurement.

Data obtained from the thermal inactivation experiments were analysed using the assumption of the first-order irreversible mechanism (Equation (1)) described by the first-order kinetic equation (Equation (2)) where *k* is the inactivation rate constant and *a* normalized enzyme activity observed in inactivation time *t*. Evaluation of inactivation data was carried out in isothermal and multitemperature approaches which were discussed in detail in [22,39,41]. In isothermal evaluation, individual inactivation curves are fitted separately to each inactivation dataset whilst in multitemperature evaluation inactivation data for all temperatures are fitted simultaneously using the temperature dependence of the rate constants expressed by the rearranged Arrhenius equation (Equation (3)). Symbols *E*, *R* and *k*_0_ in Equation (3) represent the activation energy of the reaction, the universal gas constant and the rate constant at the reference temperature *T*_0_, respectively (for conditions with and without glucose, *T*_0_ was equal to 50 °C and 35 °C, respectively).

(1) $$Active\;\overset{k}{\to }Inactive$$

(2) $$a=exp\;\left(-kt\right)$$

(3) $$k=exp\left(ln\;k_{0}\;\right)\;exp\left(\frac{E_{a}}{RT_{0}}\left(1-\frac{T_{0}}{T}\right)\right)$$

## 4. Conclusions

In this study, we investigated the catalytic stability of the S-HPED enzyme, which holds significant potential as a novel biocatalytic tool for synthesizing chiral alcohols or ketones [21]. The maximum reduction activity of S-HPED was observed at pH 5.5, where the enzyme’s inactivation and aggregation dynamics are most pronounced. The relationship between the catalytic stability and conformational stability of the enzyme across different pH levels was examined. Our findings indicate that the enzyme exhibits the highest stability in an alkaline pH environment (pH 9.0), albeit with minimal reduction activity. It has been demonstrated that the presence of glucose within the pH range of 5.5 to 9.0 significantly enhances the thermal stability of the protein by attenuating the dynamics of its aggregation and inactivation.

The mechanism and kinetics of enzyme inactivation under pH 9 conditions, where the loss of activity is solely attributable to temperature, have been meticulously characterized. It has been established that at pH 9.0, the enzyme undergoes inactivation following the “one-step, two-states” mechanism, was described by the first-order equation of inactivation kinetics. The validity of the mechanism is supported by the absence of any association/aggregation phenomena in the process of S-HPED inactivation under the specified pH conditions, which aligns with DLS observations indicating a constant and stable enzyme hydrodynamic radius throughout the duration of inactivation.

*S*-1-(4-hydroxyphenyl)-ethanol dehydrogenase, alongside *R*-1-(4-hydroxyphenyl)-ethanol dehydrogenase, represents the second enzyme within the short-chain dehydrogenase/reductase family for which the one-step thermal inactivation mechanism has been confirmed under similar pH conditions. At the current stage of research, while this similarity or convergence is noted, it does not form the basis for drawing a general conclusion about the mechanism of thermal inactivation of this class of biocatalysts. However, substantiating the first-order mechanism across a broader spectrum of enzymes within this group would furnish stronger evidence to support such a general hypothesis.

## Figures and Tables

**Table 1:** Substrates tested with S-HPED.

No.	Substrate	Product	Product S (%)	Product R (%)	% ee	Relative Activity * (%)
0	acetophenone	1-phenylethanol	100	0	100	100
1	4′-chloroacetophenone	1-(4-chlorophenyl)ethanol	100	0	100	123
2	4′-metoxyacetophenone	1-(4-methoxyphenyl)ethanol	100	0	100	82
3	4′-fluoroacetophenone	1-(4-fluorophenyl)ethanol	100	0	100	108
4	4′-nitroacetophenone	1-(4-nitrophenyl)ethanol	-	-	100	201
5	3′-aminoacetophenone	1-(3-aminophenyl)ethanol	100	0	100	57
5	4′-aminoacetophenone	1-(4-aminophenyl)ethanol	96	4	92	7
7	4′-ethylacetophenone	1-(4-ethylphenyl)ethanol	-	-	100	70
8	4′-bromoacetophenone	1-(4-bromophenyl)ethanol	100	0	100	130
9	4-acetylbenzonitrile	4-(1-hydroxyethyl)benzonitrile	-	-	100	186
10	2,2-dichloroacetophenone	2,2-dichloro-1-phenylethanol	-	-	100	344
11	4′-acetylbiphenyl	1-(biphenyl-4-yl)ethanol	-	-	100	204
12	1-indanone	1-indanol	-	-	-	0
13	2,3-benzofuran	2,3-dihydro-1-benzofuran-3-ol	-	-	-	0
14	3-phenyl-1-indanone	3-phenyl-1-indanol	-	-	-	0
15	6-hydroxy-1-indanone	2,3-dihydro-1H-indene-1,6-diol	-	-	-	0

* Activity was measured for 2 mM and 4 mM of substrate in 100 mM MES/KOH at pH 6.5 and 30 °C with 10% isopropanol in the reaction mixture. The activity with acetophenone was used as a reference.

**Table 2:** Activity of S-HPED at 45 °C in the pH range of 5.5–9.0 *.

	Time	pH 5.5	pH 6.5	pH 7.5	pH 8.3	pH 9.0
without additives	0 h	100%	100%	100%	100%	100%
	0.5 h	0.3%	3.2%	2.4%	2.5%	2%
with 2 mM acetophenone	0 h	100%	100%	100%	100%	100%
	0.5 h	1.4%	2.6%	2.4%	2.1%	1.2%
with 1.5 M glucose	0 h	100%	100%	100%	100%	100%
	0.5 h	9.3%	62.5%	38%	48%	42.3%
	2 h	3.5%	10%	8.7%	9.8%	15.6%
	5 h	2%	5.4%	3%	2.1%	9.3%

* Activity assay was performed at pH 6.5 as described in the methods section (Section 3.7).

**Table 3:** Results of isothermal evaluation of *S*-HPED thermal inactivation at pH 9.0 using the first-order model; SD—standard deviation of activity data fit; sso—residual sum of squares.

Temperature (°C)	Glucose-Free			in 1.5 M Glucose		
	*k* (min^−1^)	sso	SD (%)	k (min^−1^)	sso	SD (%)
25	0.00583 ± 0.00022	0.01418	3.303	---	---	---
30	0.01489 ± 0.00053	0.01373	3.132	---	---	---
35	0.04029 ± 0.00144	0.01639	3.305	---	---	---
40	0.10868 ± 0.00301	0.00563	2.082	0.00315 ± 0.00011	0.01119	3.19
45	0.36106 ± 0.01189	0.00155	1.488	0.00867 ± 0.00026	0.01694	3.36
50	---	---	---	0.03558 ± 0.00134	0.01179	3.274
55	---	---	---	0.12340 ± 0.00354	0.00661	2.451
60	---	---	---	0.36083 ± 0.00673	0.00296	1.57

**Table 4:** Results of multitemperature evaluation of thermal inactivation of S-HPED at pH 9.0. SD—standard deviation of data fit; n—degrees of freedom; E_a_—energy of activation; k_0_—rate constant at reference temperature T_0_ which was taken from the middle of investigated inactivation temperature range (50 °C in 1.5 M glucose and 35 °C for glucose-free experiments).

Parameters/ Inactivationconditions	k_0_ (min^−1^)	E_a_ (kJ mol^−1^)	SD (%)	n
glucose-free	0.04266 ± 0.00127	156.60 ± 4.46	3.27	65
in 1.5 M glucose	0.03447 ± 0.00127	211.55 ± 4.63	3.78	63

## Data Availability

The data presented in this study are available on request from the corresponding author.

## Supplementary Materials

The following supporting information can be downloaded at: https://www.mdpi.com/article/10.3390/ijms25137385/s1.
