# Supplementary material for: Catalytic Stability of S-1-(4-Hydroxyphenyl)-Ethanol Dehydrogenase from Aromatoleum aromaticum
Source: Int J Mol Sci. 2024 Jul 5;25(13):7385. doi: 10.3390/ijms25137385 (PMC11242144; doi:10.3390/ijms25137385)
Supplement: Supplementary file 1 [file ijms-25-07385-s001.zip › ijms-3048943-supplementary.pdf]

Supplementary Material

Catalytic stability of *S*-1-(4-hydroxyphenyl)-ethanol dehydrogenase from *Aromatoleum aromaticum*

Mateusz Tataruch[1], Viera Illeová[2], Anna Kluza[1], Patrik Cabadaj[2], Milan Polakovič[2,*]

[1] Jerzy Haber Institute of Catalysis and Surface Chemistry Polish Academy of Sciences, Niezapominajek 8, 30-239 Krakow, Poland

[2] Institute of Chemical and Environmental Engineering, Slovak Technical University, Radlinského 9, 812 37 Bratislava, Slovakia

* Correspondence: milan.polakovic@stuba.sk

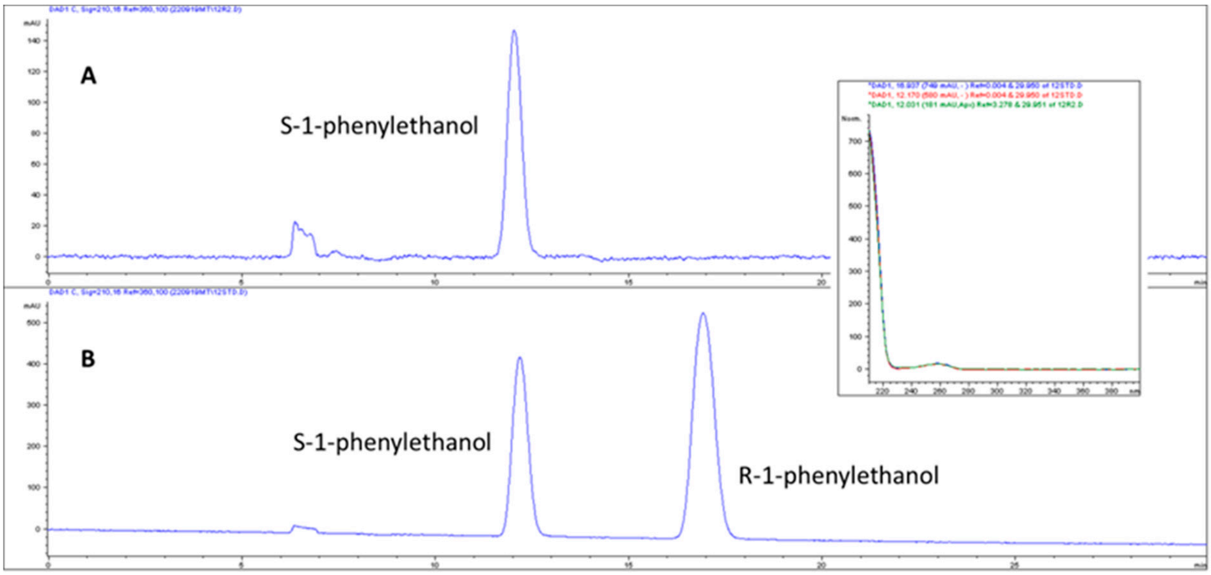

Figue S1. Chiral chromatography analysis of product formed from **acetophenone** with S-HPED. A) chromatogram of reaction mixture, B) standards of S- and R-1-phenylethanol. IPA/n-hexane ratio: 10/90.

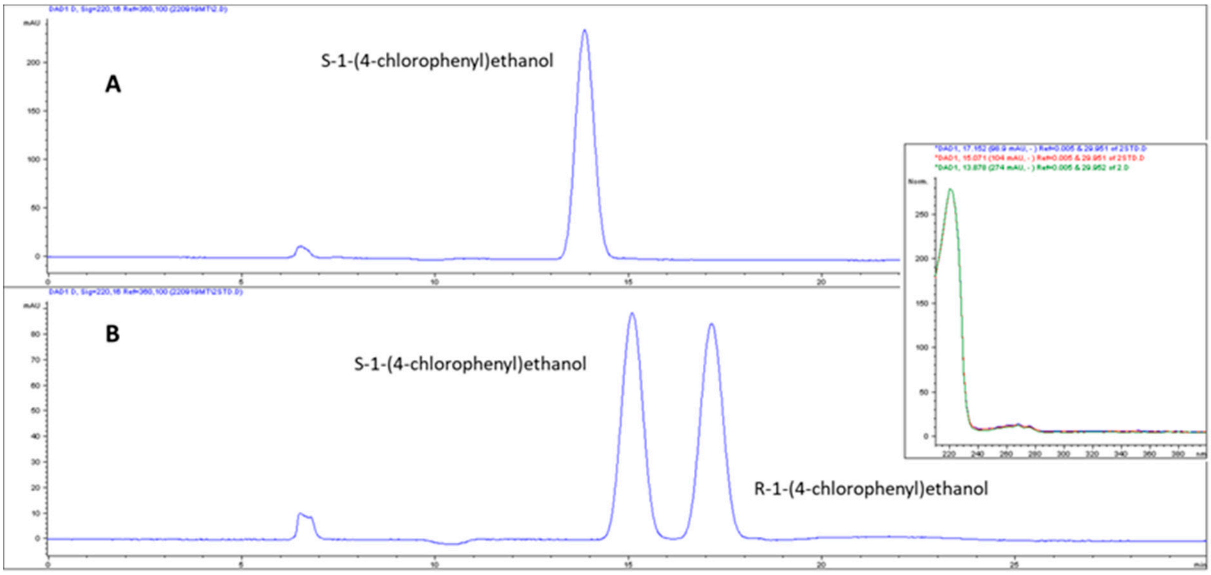

Figure S2. Chiral chromatography analysis of product formed from **4'-chloroacetophenone** with S-HPED. A) chromatogram of reaction mixture B) standards of S- and R-1-(4-chlorophenyl)ethanol. IPA/n-hexane ratio: 15/85.

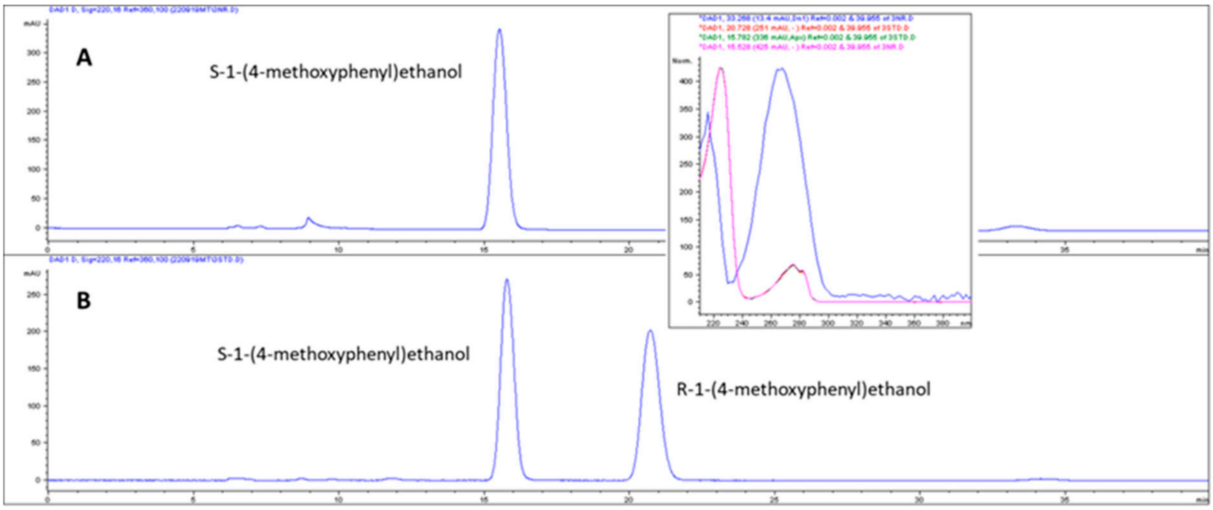

Figure S3. Chiral chromatography analysis of product formed from **4'-metoxyacetophenone** with S-HPED. A) chromatogram of reaction mixture B) standards of S- and R-1-(4-methoxyphenyl)ethanol. IPA/n-hexane ratio: 25/75.

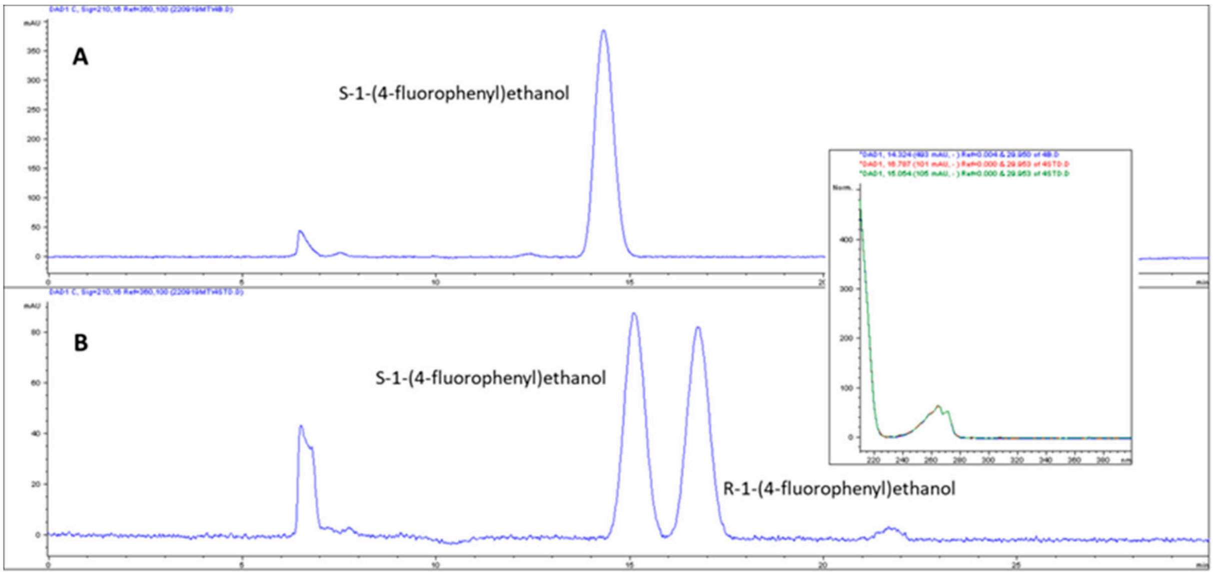

Figure S4. Chiral chromatography analysis of product formed from **4’-fluoroacetophenone** with S-HPED. A) chromatogram of reaction mixture B) standards of S- and R-1-(4-fluorophenyl)ethanol. IPA/n-hexane ratio: 10/90.

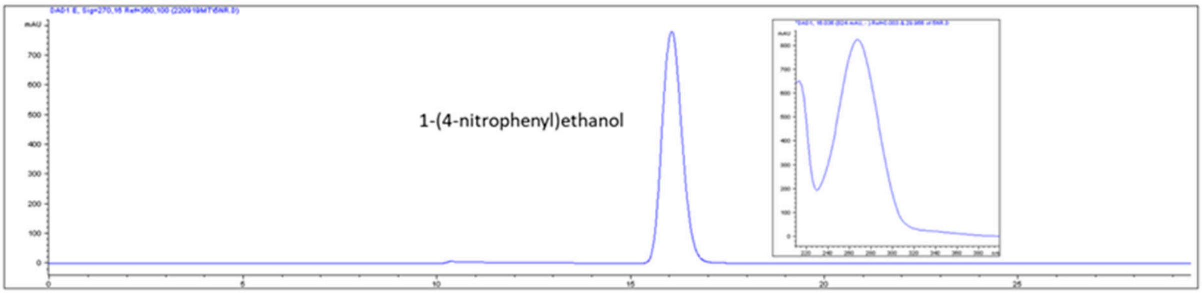

Figure S5. Chiral chromatography analysis of product formed from **4’-nitroacetophenone** with S-HPED. Chromatogram of reaction mixture. IPA/n-hexane ratio: 20/80.

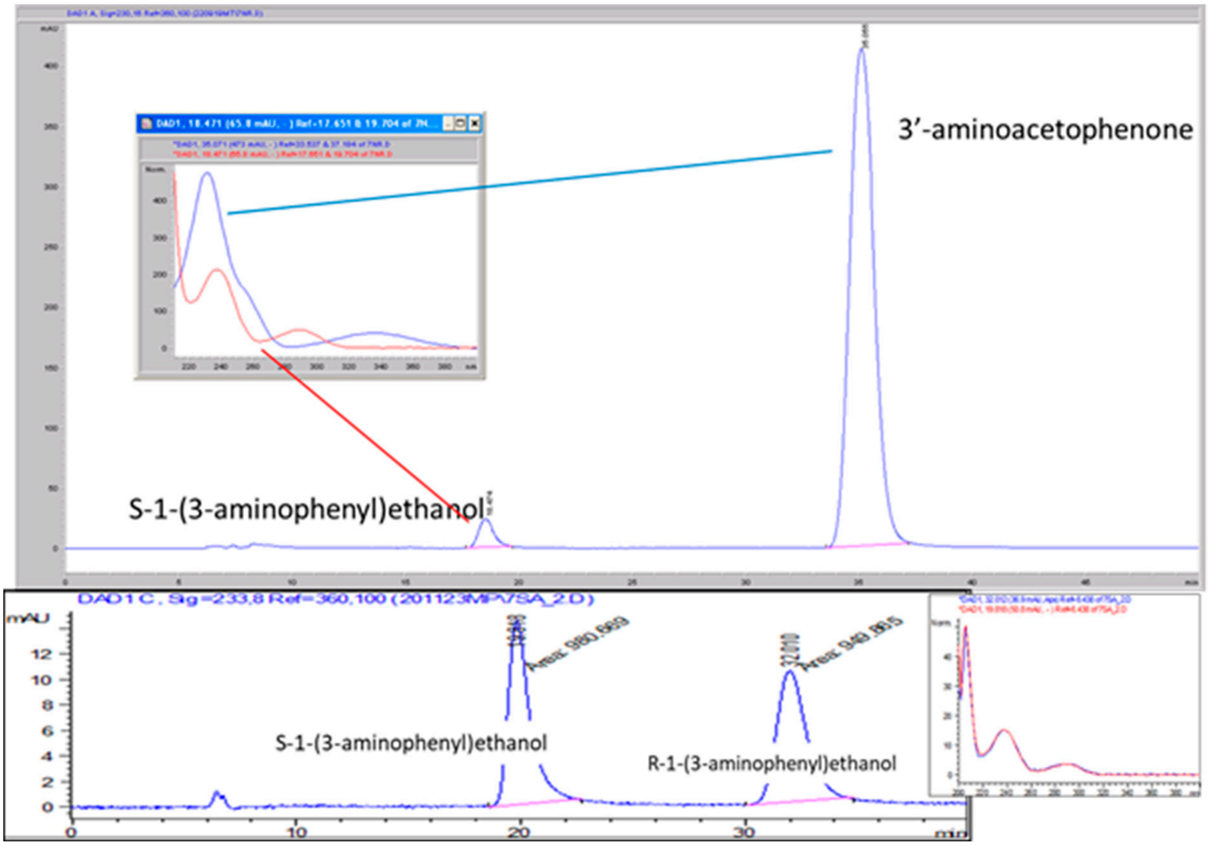

Figure S6. Chiral chromatography analysis of product formed from **3'-aminoacetophenone** with S-HPED. A) chromatogram of reaction mixture B) standards of S- and R-1-(3-aminophenyl)ethanol. IPA/n-hexane ratio: 35/65.

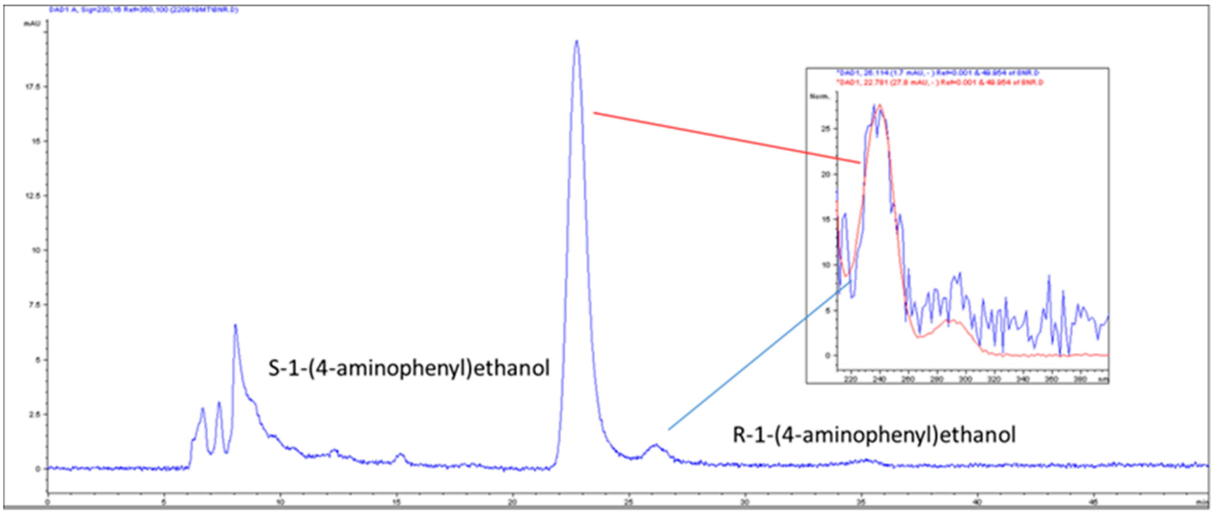

Figure S7. Chiral chromatography analysis of product formed from **4'-aminoacetophenone** with S-HPED. Chromatogram of reaction mixture with UV-vis spectrum of peaks putatively of S- (red colour) and R- (blue colour) form of product 1-(4-aminophenyl)ethanol. Retention time of products complies with retention times in [1]. IPA/n-hexane ratio: 30/70.

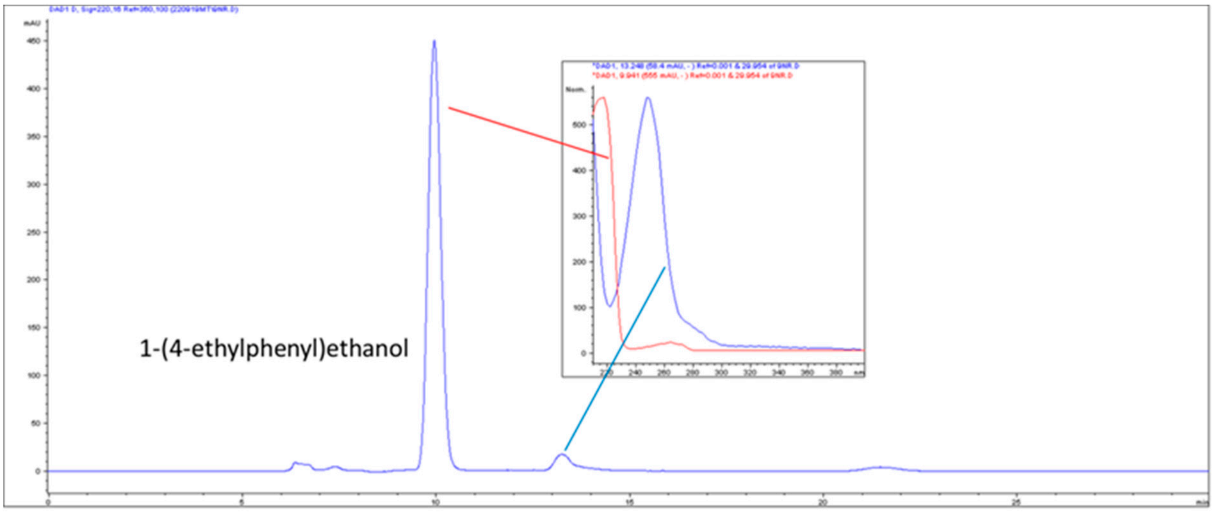

Figure S8. Chiral chromatography analysis of product formed from **4’-ethylacetophenone** with S-HPED. Chromatogram of reaction mixture with UV-vis spectrum of peaks of product and substrate. The retention time of product complies with the retention time S-form in [1]. IPA/n-hexane ratio: 10/90.

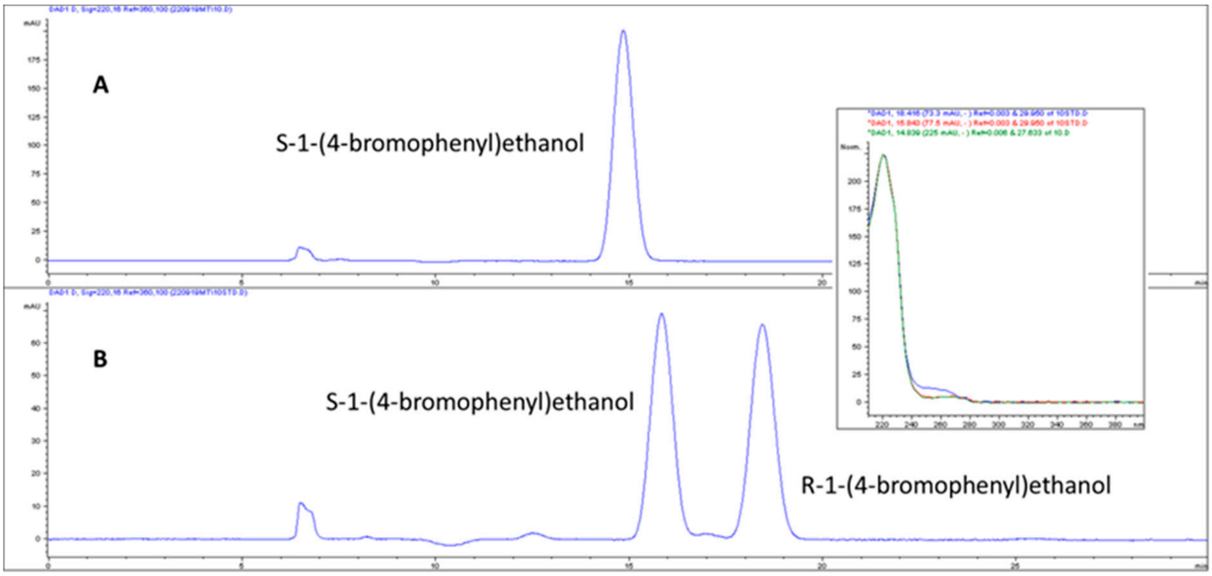

Figure S9. Chiral chromatography analysis of product formed from **4’-bromoacetophenone** with S-HPED. A) chromatogram of reaction mixture, B) standards of S- and R-1-(4-bromophenyl)ethanol. IPA/n-hexane ratio: 15/85.

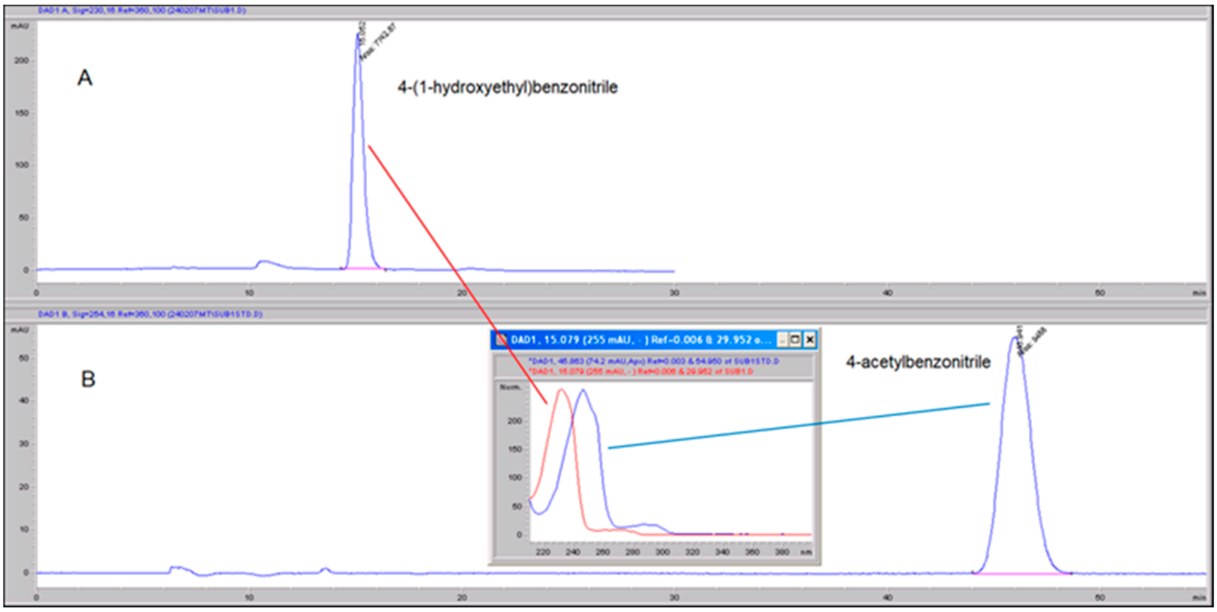

Figure S10. Chiral chromatography analysis of product formed from **4-acetylbenzonitrile** with S-HPED. A) Chromatogram of reaction mixture and pick of product, B) standards of substrate 4-acetylbenzonitrile. IPA/n-hexane ratio: 15/85.

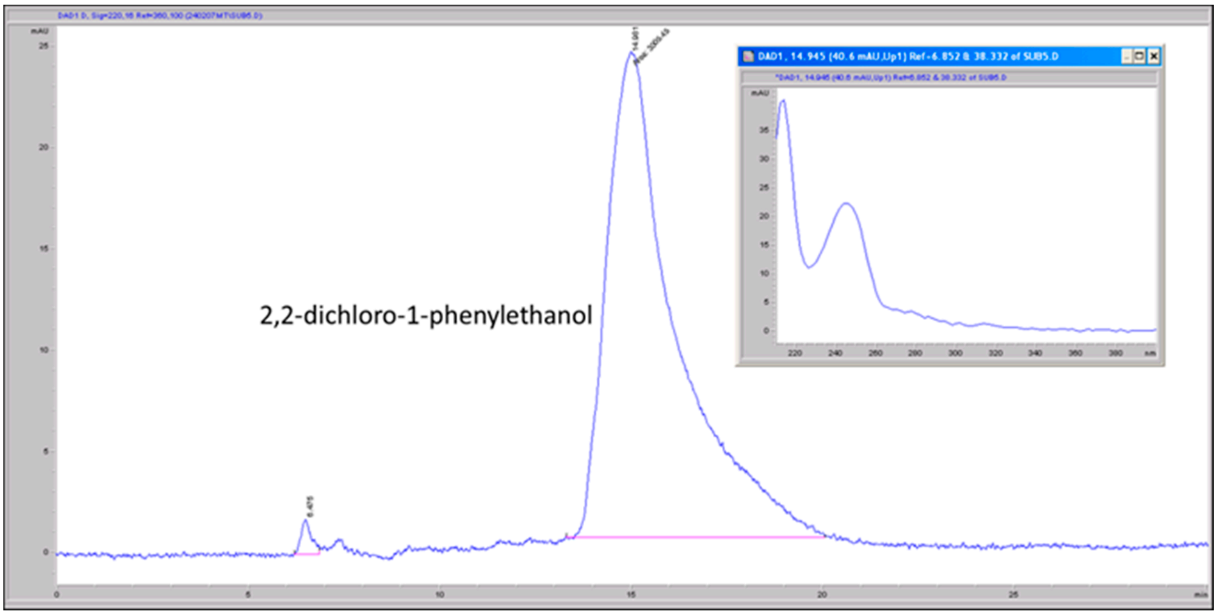

Figure S11. Chiral chromatography analysis of product formed from **2,2-dichloroacetophenone** with S-HPED. Chromatogram of reaction mixture. IPA/n-hexane ratio: 10/90.

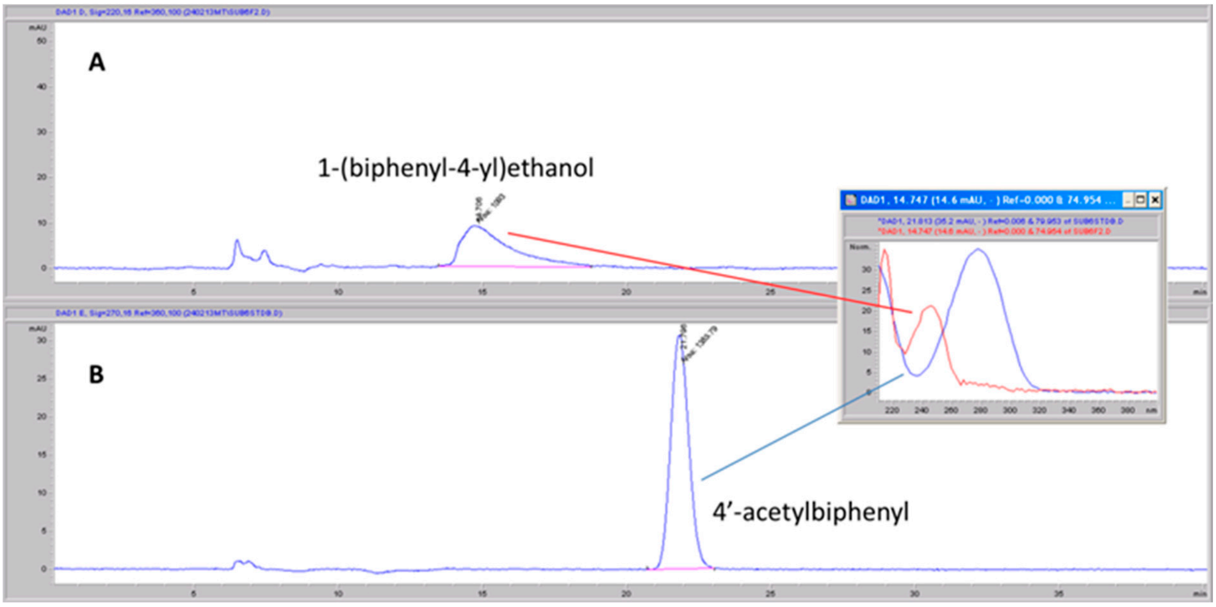

Figure S12. Chiral chromatography analysis of product formed from **4'-acetylbiphenyl** with S-HPED. A) chromatogram of reaction mixture and pick of product, B) standards of substrate **4'-acetylbiphenyl**. IPA/n-hexane ratio: 10/90.
